# Repertory of the Symptoms of the Ovaries

**Published:** 1895-12

**Authors:** C. M. Boger

**Affiliations:** Parkersburg, W. Va.


### REPERTORY OF THE SYMPTOMS OF THE OVARIES.

C. M. BOGER, M. D., PARKERSBURG, W. VA.

### MISCELLANEOUS SYMPTOMS.

Abscess of (compare Suppuration): coni. crot-h. *hep.* *lach.* (l.) *merc.* PLAT. psor. PYROGEN. sil.
Aching in (comp. Dull pains): amm-c. ars. cocaine, coni. fluor-ac. *lact-ac.* (r.) lill-t. medor. onos. ovi-gall-p. (l.) pic-ac. (l.) pod. (l.) sep. sul. syph. (l.) thuj. (l.) xanth. (r.).
Acute pain in. See Sharp.
After pains, like: kali-ph. mag-ph.
Agonizing pains in: guai.
Alternating pain between eye and ovary: sulph.
— — — ovary and groin or pudenda: plat.
— — from one to the other: colo. *lac-can.*
— with headache: ovi-gal-p. (l.).
Anæmia of: ferr. graph.
Atony of: alet. *eup-pur.* (helo.) pul.
Atrophy of: *bar-m.* CON. helo. IOD. plb.
Ball, ovary feels like a heavy b.: carb-an. (r.)
— sensation of a b. in region of ovary: calc-c.
Balls of fire, ovaries feels like: znc-val.
Bearing down in: *alo.* apis. (r.) arg-m. BELL. *canth.* cimi. ham. iod. lac-can. lac-def. lach. (l.) lill-t. mag-m. xanth. (r.).
— — in left, extending up back and down left thigh: arg-m.
Beating, sensation as if uterus were b. against right ovary: angustura. (r.).
Blenorrhœa: arn. merc. nit-ac. pul. thuj.
Boring in: brom. (l.) COLO. *lach.* LYC. lill-t. sumbul. (l.) thuj. (l.) znc. (l.)
Bruised sensation in: alet. (r.) apis. (r.) arg-m. (l.) *therid.*
Bubbling in: medor. (r.) (berb.).
Burning in: abrot. (l.) ananth-m. APIS. ARS. (r.) *bell.* (r.) buf. calc-c. canth. carb-an. colo. (r.) (con.) eupion. (l.) goss. (l.) *kali-iod.* kali-n. (r.) lac-can. LACH. (l.) lill-t. (r.) *lyc.* medor. (l.) nat-m. PLAT. sep. thu. (l.) ustl. znc. (l.) znc-val.
Burning in: paroxysms in ovaries: plat.
— heat in: buf. MEDOR. (l.)
Burst, sensation as if ovaries would: graph. (r.) medor. (l.) thu.
Cancer of: ars. graph. iod. kre. lapis-alb. psor.
Clawing in: BELL.
Coldness, sensation of, in: ferr-iod. (l.).
Colic in: apis. (r.) lach. (l.) *colo.*
Come and go, pains: goss.
— — — — quickly, pains: *bell.*
Congestion of: aco. *alet.* APIS. *bell.* bry. cact. canth. chin. cimi. con. cub. graph. ham. hedeoma. hep. iod. *kali-iod.* (r.) lac-can. (r.) lach. lill-t. magnol. (l.) melil. naj. phyt. PLAT. polygon. puls. rhus. sabi. sec-c. (r.) *sep.* staph. sulph. SYPH. thu. ustl. vibur-op. znc.
Constant pain: brom. ustl.
Constriction in (comp. Grasping): cact-g. puls. (r.).
Contraction. See Drawing.
Corkscrew pains. See Boring.
Cramping in (comp. Colic, constriction, etc.): buf. coca. (l.) coccl. *colo.* (l.) cub. (r.) naj. (l.).
Crawling in: hep.
Cutting in: abs. abro. (l.) amm-c. (l.) APIS. arg-n. (r.) ars. aru-t. (l.) ATROP. (l.) BELL. (r.) bor. bry. canth. *coccl.* cocc-c. collin. (r.) COLO. CON. cub. (r.) eup-pur. (l.) fluor-ac. graph. (l.) ham. lach. LILL-T. lyc. medor. naj. nat-m. nux-m. onos. phos. (l.) pul. (l.) *sabad.* stram. syph. (l.) thu. ustl. (l.) xanth. (r.).
Cysts of: apis. *bov.* canth. carb-an. *colo.* IOD. *kali-br.* lach. murex. prun-sp. PLAT. rhod. (r.) rhus-t. thu.
Dark color of: ars.
Darting in: abro. (l.) abs. (r.) *bell.* graph. lac-can. (r.) lach. (l.) lill-t. MAG-PH. (r.) phos. sep. (r.) syph. thu. xanth. (r.).

MISCELLANEOUS SYMPTOMS.

Debility of: chenop-v.

Dislocated, pain as if, in (comp. Shaking loose): apis.

Disorganization of: ARS. sec-c. (r.).

Distention of (comp. Enlargement, etc.): aur-mur-nat.

— sensation of: curar. medor. (l.).

Distressing pain in: lill-t. (pho.) thu. (l.) ustl.

Dragging in: bry. (r.) lac-def. (l.) lac-fel. (l.) lill-t. (r.) (oxalis). plat. (l.) pod. sil. (l.).

Drawing in (comp. Pulling, etc.): apis. ars. (r.) atrop. (l.) bell. (r.) chin. cocc-c. colo. (l.) cub. goss. lach. (r.) lill-t. (l.) PLAT. pod. (r.).

— — toward uterus: goss.

— downward and forward: pallad. (r.).

Drawn together, sensation as if heart and ovary were: naja.

Dropsy of: APIS. arn. *ars.* *aur-m-n.* bell. brom. (l.) bry. calc-c. carb-an. chin. colo. con. ferr-iod. (r.) graph. *iod.* kali-bro. kali-c. kre. lach. LILL-T. LYC. merc. nat-s. phos. plat. plb. pod. pru-sp. rhod. rhus-t. sabin. sep. staph. tereb. znc.

Dull pain in (comp. Aching): amm-br. (l.) apis. (r.) brom. (l.) carb-v. (l.) con. *ferr-ph.* hydrocot. iod. kre. lill-t. (l.) sep. sulph. xanth. (r.).

— — — left and in spleen: carb-v.

Ecchymosis in: merc-corr.

Enlarged sensation in: ARG-M. (l.) *arg-n.* (r.) medor. (l.).

Enlargement of (comp. Distention, swollen, etc.): APIS. (r.) *aur-m-n.* (r.) *bell.* (r.) CON. *graph.* (l.) *iod.* hep. kali-br. lac-can. (l.) LACH. (r.) lill-t. (l.) lyc. melil. (l.) spo. ustl. (l.).

Fallen, pain in left as if it had f.: medor. (comp. Rolled over).

Fire: balls of: ovaries feel like: znc-val.

Fleeting pains in: curar.

Fluttering in: brachy. (r.).

Fullness in: syph.

Gnawing in: colo. (l.) lill-t. (r.).

Grasping in: (cact-g.) *lill-t.* (l.) oxytropis (l.).

Grinding in: graph. (r.) fluor-ac. (r.).

Griping in (comp. Colic): curar. (r.) lill-t.

Hardness of (comp. Induration): amm-br. amyl-n. APIS. (r.) (ars.) BELL (r.) brom. (l.) carb-an. graph. (l.) lach. (l.) *ustl.* (l.).

Heat in (comp. Burning): *bufo.* lac-can. lac-def. med. (l.).

Heaviness of (comp. Weight): APIS. (l.) ars. carb-an. (r.) carb-v. (l.) eup-pur. (l.) helo. iod. kali-c. lill-t. (l.) melil. onos. sep. vibur-op.

Heavy pains from ovarian region to shoulder-blade, with gastralgia (after menses): borax.

Holding, as if something were h. ovary. oxytrop.

Hernia of: coccl. con. mag-m. nux-v. sil. sul.

Hydatids of: *bufo.* canth. merc.

Hypertrophy of (comp. Swollen, etc.): *carb-an.* iod. lactuca. puls.

Increasing and decreasing gradually, pains: STANN. xanth.

Induration of (comp. Hardness, etc.): amm-br. (l.) apis. aur-m-n. ars. *bar-m.* bar-iod. *bell.* brom. carb-an. (r.) *con.* curar. GRAPH. (l.) IOD. LACH. (l.) *pallad.* (r.) plat. psor. (l.) *sep.* spon.

Inflammation (comp. Congestion): aco. æsc. (r.) amm-br. amb. ant-c. APIS. (r.) arg-m. arn. ars. aur. BELL. (r.) brom. bry. cact. canth. caps. (l.) *chin.* CIMI. colo. con. crot-h. cub. dulc. euphorb. gel. graph. (l.) guai. ham. hedeom. igt. iod. (r.) kali-iod. lac-can. *lach.* (l.) lill-t. LYC. (r.) mag-pho. *merc.* nit-ac. nux-v. *pallad.* (r.) phos-ac. PHOS. phyt. plat. POD. (r.) *puls.* rhus-t. sabad. SABIN. salix-n. staph. syph. thu. (l.) USTL. *verat-v.* znc. (l.).

— chronic of: brom.

Insupportable pain in: alumen (l.).

Intense pain in: ars. xanth. (l.).

Intermittent pains in (comp. Periodical): goss. lac-can. (r.) ustl. (l.) ziz.

Irritation of: *amm-br.* APIS. bry. (r.) carb-ac. (l.) CIMI. *gels.* *ham.* *kali-br.* lill-t. nux-v. phyt. plat. rhus-t. (r.) thu. ustl. VIBUR.

Itching in: prun-sp.

Jerking in and above: eupat-pur. (l.).

Labor like. See Bearing down.

Lancinating. See Cutting.

Left ovary: abrot. æsc. agar. alumen. amb. *amm-br.* *apis.* ARG-M. arg-n. artem. aru-tri. *atrop.* bov. BROM. *caps.* *carb-ac.* carb-v. cenchris. cham. cimi. COLO. cup-ar. eup-pur.

DIRECTION OF PAIN.

eupion. ferr. fer-iod. fer-pho. *goss.* GRAPH. *ham.* hydras. iod. kali-bro. kali-ph. lac-can. lac-def. lac-fel. LACH. LILL-T. lyc. lyss. magnol. MEDOR. melil. MERC. murex. *naj.* nat-m. onos. ovi-gal-p. pic-ac. *phos.* PLAT. pod. psor. puls. rhus-t. sep. sil. stan. stram. sumbl. SYPH. therid. THU. USTL. *vesp.* wyeth. XANTH. *znc. ziz.*

Lightning-like pains in: mag-ph.

Lump in, as if: apis. (l.) pso. (comp. Swelling, etc.).

Movement in: apis.

Needles, as if in (comp. Sticking): colo.

Neuralgia of: Amm-br. amm-m. apis. (r). ATROP. *bell.* (r.) cham. CIMI. (l.) coccl. collin. COLO. *crot-h.* ferr-ph. gel. *ham. igt.* KALI-BR. kali-ph. lac-can. LACH. (l.) lill-t.(l.)LYC. MAG-PH. (r.) medor. naj. (l). pallad. (r.) phyt. plat. puls. *ran-b.* SAC-LAC. (r.) sep. stann. staph. stram. tarent. therid. urt ur. ustl. (l.) vesp. xanth. (l.) zinc. (l.) zinc-val. ziz. (l.).

Numbness in: apis. (r.) pod. (l.).

Obliterated, almost: carbon-sul.

Oppressive pain in: lill-t. (r.) melil.

Pain in (undefined): acon. æsc. (l.) alu. apis. (r.) *arg-m.* (l.) arg-n. ars. atrop. aur. BELL. (r.) berb. brom. (l.) bry. (r.) cact. calc-c. canth. carb-ac. (l.) carb-v. cenchris. cimi. collin. colo. (l.) con. cop. ferr-ph. (l.) gels. graph. (r.) guai. *ham.* hell. (r.) *helo.* hydrocot. iod. (r.) *kali-br.* (l.) kali-ph. lac-can. lach. (l) lill-t. (l.) LYC. (r.) lyss. (l.) mag-ph. medor. (l.) naj. NAT-M. (r.) onos. ovi-gal-p. (l.) pallad. (r.) phos. (l.) plat. (l). plb. pod. (r.) rhod. rhus-t. saba. sars. sec-c. sep. (l.) sil. (l.) staph. stilling. sul. syph. (r.) therid. (l.) thu. (l.) UST. (l.) vesp. (l.) vibur. wyeth. (l.) XAN. znc. (l.).

Pain over the ovaries: phos.

**Pain, direction of:**

— extending across lower part of ABDOMEN from region of ovaries: CIMI. lac-can. (l.) lill-t. (l.).

— — up to ABDOMEN from left ovary: ham. lill-t.

— — through ABDOMEN, hips, and back: coni.

— — to ARM: lac-can. (l.).

— — through small of BACK from left ovary: æsc. plat.

Pain extending up BACK from: arg-m. (l.).

— — to BACK: abrot. arg-n. brom-(l.) iod. sul. xanth. (r.).

— — — — and *abdomen:* phos.

— — BACKWARD: bell. sep.

— — to entire BODY: dios.

— — into BOWELS: cocaine.

— — to CHEST from ovaries: apis. (r.) lach. (r.) murex. (r.).

— — to left CHEST from ovary: APIS. (r.).

— — — CRURAL region and thighs: staph.

— — — — nerve (anterior): xanth.

— — — — — (descending it): pod. (r.).

— — DIAGONALLY: apis. (r.) medor. (r.) murex.

— — DOWN sides of hypogastrium: lill-t.

— — DOWNWARD: medor.

— — — and forward from left ovary: arg-m. (l.).

— — to EXTREMITIES: ustl.

— — — FOOT: lac-can. (l.). ovi-gal-p.

— — — GENITALS from ovary: lach. (r.).

— — — GROIN: amm-c. (l.) apis. (r.) BUFO. cub. lill-t. medor. (l.) thu. ustl.

— — — — left: medor. (l.) ovi-gal-p.

— — — — right: lill-t.

— — — — and hypogastrium: xanth.

— — — — — iliac region: thu. (l.).

— — — — — left leg: lill-t. thu. ustl.

— — — — — loins: cubeb.

— — — — — pubes: lill-t.

— — — — thigh and leg: ovi-gal-p. (l.).

— — through GROIN toward: sacrum: PLAT.

— — to HEART from ovary: brom. dios. lill-t. ovi-gal-p.

— — like a drawing from ovary to HEART: *naja* (l.).

— — HIPS: apis. (r.). *brom.* (l.). bry. merc. xanth. (r.).

— — down HIPS: ustl. (l.).

— — through HIPS to back: æsc. (r.).

— — to HIPS and thighs: apis. lill-t.

— — over HIPS and down thighs: xanth. (r.).

— — — right HYPOCHONDRIUM: lill-tig. (r.) lach. (r.).

DIRECTION OF PAIN.

Pain extending across HYPOGASTRIUM, to groin and down leg: lill-t.
— — to HYPOGASTRIUM: lill-t. xanth.
— — through ILIAC region, into groin and sometimes into left leg: thu. (l.).
— — over ILIUM, either to or from ovary: SEP.
— — to KNEE (shooting): cimi. lac-can. (r.). wyeth (l.).
— — from LEFT to RIGHT: apis. ip. lac-can. LACH. lill-t. naj. syph. ustl.
— — to LEG (left): apis. cham. lac-can. lill-t. ovi-g-p. phos. thu. ustl.
— — — — — — and foot: ovi-gal-p.
— — — — — (right): apis. LYC.
— — — — — (shooting down): ustl. (l.).
— — — LIVER: lach. (r.) *lill-t.* (r.) med. (r.).
— — down LIMBS: ferr-iod (l.) goss. (l.).
— — to LOINS: staph.
— — — — and groins: cub.
— — — LUMBO SACRAL region: lill-t.
— — — left MAMMARY region: lill-t. (l.) murex. (r.).
— — — opposite MAMMARY gland: murex.
— — from ONE to the OTHER: cimi. ONOSMOD.
— alternating from ONE to the OTHER: LAC-CAN.
— EXTENDING, OUTWARD and backward: sep.
— — to OVARIES, from back, over each hip: SEP.
— — — OVARY from groin: alumen. (l.).
— — — — — — heart: *ovi-gall-p.* (l.).
— — — — — — left labium through uterus: bell. phos. thu.
— — — — — — left leg: lac-can. (l.).
— — — — — — lumbar region: tereb.
— — — — — — region of epigastrium: ovi-gal-p. (l.).
— — — — — — sacral region: ustl. (l.).
— — — — — — umbilicus: colo. hydrast. (l.) (pallad.).
— — into PUBES: cocc-c.
— — across PUBES from ovaries: lill-t. (l.).
— — to RIBS: apis. (r.).
— — from RIGHT to LEFT: *bell.* graph. LYC. xanth.
— — SACRUM and thighs from: arg-n.
— — SHOULDER-blade from: borax (r.).
Pain extending to SHOULDER from ovary: pod.
— — up to SIDE from ovary: cimi.
— — to whole left SIDE: lac-can. (l.) plat. (l.).
— — from SIDE to SIDE: cimi.
— — toward STOMACH: colo. (l.).
— — to THIGHS: amm-c. (l.) APIS. arg-m. argn-n. ars. bry. (r.) *cact. calc-c.* (r.). carb-an. cham. lac-can. lill-t. (l.). magnol. (l.) nat-m. (r.) ovi-gal-p. (l.) pallad. (r.) phos. pod. staph. xanth.
— — — — *anterior* surface of: lill-t. (l.) xanth. (l.).
— — — — *inner* surface of: ars. (r.) lill-t. (l.) phos. (l.).
— — — — — — — — and down knee: pod. (r.).
— — — — *outer* surface of: lill-t.
— — — — *posterior* surface of: ars. ovi-gal-p. phos.
— — — — knee and foot: ovi-gal-p.
— — — — and crural region: staph.
— — — — — — over hip, from ovary: xanth.
— — UPWARD (shooting): arg-m. cimi. lac-can. lill-t. lach. pod.
— — through UTERUS from ovaries: bell.
— — to UTERUS: cubeb. goss. ham. (r.) helo. iod. (r.) lach. (r.) sac-lac. (r.) sep. ustl. (l.).
— — — — and back: ustl.
— — — — through broad ligament: ham. iod. (r.).
— — — — — — ovarian region: medor.
— — — VAGINA from: ovi-gal-p. (l.). sep. (l.).
— — EXTENDING into VESICAL and pubic region from ovaries: *cocc-c.*
Paroxysmal pain in: apis. lach. plat.
Periodical pains in: *apis.* cact-g.
Pinching pains in: *canth.* cham. PLAT.
Plug, as if a dull p. were driven from right ovary toward womb: iod.
Pressive pains in: ars. (r.) iod. lac-def. LACH. (l.) plat.
Pressure in: angust. (r.) carb-v. (l.) sep.
— above: eupat-pur. (l.).
— downward: plat.
— — on: lill-t.
Pulling-down sensation in (comp. Drawing): cub. goss. medor. (l.) (pallad.) plat.
Pulsation (including throbbing, etc.) apis. ars. BELL. (r.) CACT-G. calc-c.

DIRECTION OF PAIN.

cop. cub. cup. onos. nux-m. pod. (r.).
Pulsation, at same hour every day, extending to thighs: cact-g.
Pushing in: caps. (l.).
Quick pain: bell. eupat-pur. (l.) (comp. Cutting).
Rawness, sensation of: merc. (l.).
Rending. See Tearing.
Right ovary: abs. ÆSC. angust. APIS. arg-m. *arg-n.* *ars.* BELL. bor. brachy. *bry.* calc-c. carb-an. cenchris. collin. conval. *colo.* cop. cub. curar. ferr. fer-iod. fluor-ac. glon. graph. ham. hell. iod. kali-bro. *kali-iod.* kali-n. lac-can. *lach.* lact-ac. *lill-t.* LYC. mag-ph. medor. murex. nat-m. PALLAD. pic-ac. plat. POD. pso. puls. rhod. rhus-t. sac-lac. sars. sec-c. sep. syph. *xanth.*
Rolled over, sensation as if ovary (comp. Fallen): lach. lill-t. ovi-gal-p.
Sensitiveness, including soreness and tenderness: alumin. (agar.) ANT-C. APIS. (r.) arg-m. arg-n. ARN. artem, atrop. (l.) BELL. bry. (r.) bufo. calc-c. canth. chin. cimi. colo. (l.) con. cup ars. (l.) *graph.* guai. ham. helo. hep. *iod.* (r.) jecoris. kali-br. (l.) lac-can. LACH. LILL-T. LYC. medor. (l.) murex. (r.) NUX-M. *onos.* ovi-gal-p. (l.) PALLAD. (r.) *plat.* (l.) psor. (r.) puls. rhus-t. salix-n. sec-c. (r.) sep. STAPH. syph. (l.) tarent. *tereb.* thea. therid. ustl. (l.) vesp. (l.) znc-val.
Scirrhus of: ars.
Severe pain in: bry. (r.) ovi-gal-p. ustl.
Shaking loose, as if something were in region of ovary: lill-t.
Sharp pain in (comp. Cutting): abrot. absin. (r.) APIS. (r.) curar. cenchris. (l.) colo. fluor-ac. hydrast. (l.) kali-ph. (l.) *lac-can.* (r.) LACH. (l.) LILL-T. lyc. medor. naj. onos. sep. STAPH. syph. (l.) ustl. (l.) verat-vir. vibur-op. xanth. (r.).
Shooting in: abrot. (l.) absin. (r.) apis. (l.) brom. bry. cenchris. (l.) cub. graph. LACH. (l.) *lill-t.* (l.) lyc. MAG-PH. (r.) medor. merc. pod. (r.) sac-lac. staph. thu. (l.) VIBUR. xanth. (r.).
— across pubes: lill-t. (l.)
— over ovaries: vibur.
— to knee: wyeth. (l.).
— — hip: xanth. (r.).
Shooting up side: cimi.
— — left side and down arm: lac-can.
Smarting (comp. Rawness): eup-pur. (l.) syph. (l.).
Soreness. See Sensitiveness.
Sore spot, pain as of a, in region of right extending to thighs: *bry.*
— — in left: ovi-gall-p. syph. (l.).
Spasmodic pain in: colo. (l.) mag-ph. phos. ustl. (l.).
Sprained, as if. See Strained.
Squeezing pain in (comp. Grasping, etc.): COLO. (l.) (staph.) thu. (l.)
Stabbing in colo: saba.
Steady pain in: apis.
Sticking in (comp. Needles), etc.: caps. (l.) colo. (graph.) *plat.* (r.) *sep.* (l.).
— from cervix to fundus of uterus, and then to right ovary, causing nausea: sepia.
Stinging pains in: APIS. (r.) bor. bry. goss. graph. lill-t. (l.) merc. sep. thu.
Stitches in: AMB. ARS. (r.) BELL (r.) bor. brom. *bry.* *bufo.* canth. *colo.* (r.) carb-an. con. curar. graph. kali-c. LACH. (l.). LILL-T. *lyc.* merc. (l.) ovi-gal-p. (l.) sep. staph. thuj. ustl.
Strained, pains as if: *am-m.* apis. (l.) ARN.
Sudden pain: bell.
Suppuration. See Abscess.
Swelling of (comp. Enlargement): ananth-m. *alu.* amm-bro. (l). APIS. (r.) ARS. (r.) ATROP. (l.) BELL. BROM. (l.) buboin. (l.) *bufo.* CARB-AC. (l.) cenchris. collin. colo. (l.) con. cub. goss. GRAPH. (l.) ham. IOD. KALI-BRO. (l.), KALI-IOD. (r.) LACH. (l.) LILL-T. (l.) nux-m. PALLAD. (r.) staph. syph. (l.) thu. *ustl.* (l.).
— sensation of: kali-iod. *lill-t.* (l.).
Tearing in: abrot. (l.) *graph.* (r.) *ham.* *kali-iod.* (r.) lill-t. (merc.) pallad. (r.) *plat.* (l.) thu.
Tenderness. See Sensitiveness.
Tension in both ovarian regions, as if a thread were drawn in that direction: cinnam.
Tensive pain: amm-c. (l.) ant-c. ARS. cinnam. *colo.* curar. eup-pur. lach. (l.) medor. (r.) PLAT.
Thread, sensation of a: cinnam. sep.
Trobbing. See Pulsating.
Thrusting pain in. See Cutting.
Tickling sensation in: pru-sp.

Tightness (comp. Tensive): apis. PHOS. (l.).
Tired sensation in: pod. rhus-t.
Torn sensation in: bry. (r.).
Transient pains in: abrot. abs. (r.) bell. (r.).
Tumor of: APIS. apocy-c. ARS. bar-c. BAR-M. calc-c. carb-an. colo. *con.* fluor-ac. (r.): GRAPH. hep. iod. kali-br. LACH. LYC. merc. PLAT. plb. POD. (r.) PSOR. rhus-t. staph. stram. syph. thu. znc.
— encysted, hard: carb-an.
— — soft: apis.
Twinges of pain in: pic-ac. (l.).
Twisting or wringing: graph. (r.) kali-iod. (r.).
Twitching in: kali-iod. (r.)
— — extending to back: abrot.
Uneasiness in: lyss. (l.).
Vise, as if in a: colo. (l.).
Vitality, absence of in: chenop-v.
Wedge-like pain from right ovary to uterus: *iod.*
Weight in (comp. Heaviness, etc.): APIS. con. carb-v. (r.): fluor-ac. kali-c. (l.) lac-def. (l.) *lach.* lill-t. (l.) sep.
Worrrying pain in: fluor-ac.
Wrenched as if: bry. (r.).

### Aggravation.

Abortion from: APIS. colo. ham. lact-ucarium. SABINA.
Absence of sexual intercourse: staph.
Afternoon: sep.
Alcohol: nux-v.
Anger: colo.
Anxiety, mental: ars.
Bending: ars.
Breathing: bell. *bry.* graph. lac-can. lill-t.
Bruise from: psor.
Change of weather: ran b. rhod. rhus-t.
Climacteric: LACH.
Clothes, weight of: *lach.*
Coition, from: apis. buboin. (l.) CHIN. plat. pod. syph. (l.).
Cold: acon. ars.
— dry air: acon.
— taking: graph.
Confinement, after: LACH.
Coughing: graph.
Conversation, animated: pallad.
Daytime: eupat-pur.
Debility: *phos-ac.*
Drawing in of abdomen: amb.
Emotions: igt. lach. *phos-ac.*
Evening: apis. lill-t. lyc. (4–8) pic-ac.
— and night: lill-t. merc.
Excitement: pallad.
Exertion: artem. eupion. lact-ac. lach. ovi-gall-p.
Food, highly-seasoned: *nux-v.*
Fright: aco. lyc.
Gonorrhœa: cub. ham. thu.
Heat: apis. LACH. vesp.
Hawking: graph.
Hemorrhage: chin.
Indigestion: colo. nux-v.
Injuries: *arn.* con. ham. hyper. psor.
Inspiration: bry. graph. lac-can. lill-t. sep.
Jarring: BELL. lill-t.
Love, disappointed: staph.
Lying on back: *goss.*
— — left side: calc-c.
— — right side: apis. lach.
Masturbation: staph.
Menses, beginning of: apis. *bell.* coccl. LACH. nux-v.
— before: *apis.* arg-n. brom. goss. graph. kali-n. lac-can. LACH. nat-m. naja. nux-v. phos. pod. rhus-t. thu. vibur. znc.
— during: acon. ant-c. *apis.* arg-n. atrop. bell. bor. brom. bry. canth. cham. cenchris. coccl. collin. colo. con. fer-iod. gels. goss. graph. ham. iod. kali-c. kali-n. lac-can. lach. lill-t. nat-m. pallad. phos. plat. pod. rhus. saba. sabin. sars. staph. sul. thu. ustl. xanth. znc. znc-val.
Menses after: bor. cup. goss. graph. iod. pallad. znc.
— suppressed: aco. ant-c. apis. cimi. lach. pul.
— — by bathing: ant-c. pul. sulph.
Mental agitation: pallad. phos-ac.
Midnight, at: ars.
Mind dwelling on sexual gratification: staph.
Morning: iod. lach.
Motion: amm-c. (l.) apis. ars. bell. BRY. cenchris. (l.) lac-can. lill-t. lach. pallad. therid.
— of limbs: ars. med.
Music: pallad.
Night: ars. lill-t. *merc.* pod. syph. (r.).
Periodically: ars. chin. cact.
Perspiration suppressed: *aco.*
Pregnancy, during: ham.
Pressure: amb. apis. guai. ham. kre. lac-def. lach. lill-t. medor. nux-mos. onos. sep. staph. therid. ustl.

AGGRAVATION, AMELIORATION, AND CONCOMITANTS.

Pressure of clothes: LACH.
Raising arms: apis. *graph.*
Reading: nat-m.
Riding: arg-m. thu.
Sewing-machine, running the: pod. znc.
Sexual excesses: chin.
Sexual intercourse: absence of: staph.
Sleep, after: lach.
Society, after being out in: pallad.
Standing: apis. cop. lill-t. medor. pallad.
Stimulants: nux-v.
Stooping: apis. lach.
Stretching or straightening up: apis. colo.
— — — out limbs: pod.
Strain from: rhus-t.
Strangury: canth.
Touch: alu. ant-c. apis. artem-v. bell. bry. calc-c. canth. CHIN. colo. graph. ham. iod. kali-br. lach. lill-t. pallad. sec-c. ustl.
Urinating: lyc. nat-m. (sars.).
Walking: agar. apis. ars. carb-ac. (l.) lac-ac. lach. lill-t. med. onos. ovi-gall-p. pod. thuj. ustl.
Wet, getting: aco. ant-c. pod. pul.
— — feet: graph. pul.
Windy weather: rhod.
Writing: nat-m.

### Amelioration.

Bending double: amm-m. apis. arn. colo. fluor-ac. kali-ph.
Counter irritation: lill-t.
Flatus, passage of: pod.
Holding with hands: lill-t.
Hot applications: ars. hyper. mag-ph.
Lying: onos. pallad. thu.
— on back: kali-ph.
— — face: medor.
— — left side: pallad.
— — right side: apis.
Menses, free flow of: lach. naj. nux-v.
— during: thu. znc.
Motion: iod. rhus-t.
— of feet: ars. znc.
Pressure: colo. fluor-ac. lill-t. medor. pod. znc.
Rubbing: lill-t. pallad.
Shaking: znc.
Sitting: lill-t.
Stretching, cup.
Urinating: lyc. sep.
Vaginal discharges: lac-can. lach.
Walking in open air: naja.
Warm drinks: colo.
Warm hand over: lill-t.

### Concomitants.

Abdomen, bruised soreness in walls of: APIS.
— pain in: *colo.* cup-ars. ham. nat-m.
— sensation of something rolling over in: ovi-gall-p.
Abortion: APIS. *bry.* colo. ham. hep. pod.
Alive, sensation internally: thu.
Amenorrhœa: alet. ant-c. *apis.* bar-m. bell. con. helo. *ferr.* graph. lach. lill-t. phyt. pod. ustl. xanth.
Anæmia: *ferr.* ham. *kali-permang.* PULS. therid.
Appetite ravenous with emaciation progressing: iod.
Back, cutting in: *arg-n.*
— pain in: arg-n. brom. helo. hep. iod. lill-t. sep. ustl.
— weakness of: abrot.
Bearing-down pain: BELL. curar. ferr-ph. ham. iod. lill-t. onos. pallad. plat.
Bend forward, is compelled to: *acon.* apis. arn. atrop. COLO.
Bladder, irritability of: guai.
— pressure in: carb-v.
Breast, pain in: lill-t.
Breasts, engorged: onos.
— sensitive: ovi-gal-p.
Breathing, arrest of: canth. fluor-ac. xanth. (r.)
Bronchitis: lach.
Burning sensations: ARS.
Chest, left-side, pain in: *apis.* pul.
Chilliness: puls.
Climaxis: crot-h. helo. LACH. plat. pul. ustl.
Chlorosis: artem-v. bar-m.
Chorea: cimi.
Confinement: bry.
Coition, aversion to: graph.
— absence of desire for: kali-br.
— without pleasure: berb. helo. hep. onos.
— ungratified: kali-bi. staph.
Colic: COLO.
Constipation: ALUMEN. apis. graph.
Convulsions, hysterical: stram.
Cough: alumen. *apis.* naja. phos.
Despondency: cimi. igt.
Dizziness: xanth. (r.).
Dyspnœa: dios.
Dropsy: APIS. lyc.
Dysmenorrhœa: acon. *apis.* cimi. ferr-

CONCOMITANTS.

ph. guai. ham. tarent. therid. vibur. xanth.
Dysmenorrhœa, membranous: brom. cycl. lach.
Emaciation: *iod.*
— from below upwards: abrot. arg-n.
— — above downward: lyc.
Epilepsy: atrop. kali-br. LACH. puls.
Erotomania: coca. *hyos.* plat. znc.
Eyes, sensation of soreness in: cimi. eupat-p. *onos.*
Face, flushing of: ferr.
Fear: acon.
Feet, restlessness of: ars. lac-can. *znc.*
Fever: acon. verat-v.
Forehead, stitches in: plat.
Glands, swelling of: brom.
Gonorrhœa: cop. ham. merc. plat. thu.
— suppressed: canth.
Hæmophila: crot-h. phos.
Head, aching of: atrop. ferr-ph. gel. xanth.
— — constrictive: tarent.
— congestion to: apis. BELL. iodof. tuberculin.
— forehead, supra-orbital pain, left side: lac-can.
— temple, pain in: naj. (l.).
— vertex, cutting or stitching in: arg-n.
— — pressure in: crot-h.
Hemorrhage: amm-br. chin.
Heart symptoms: lill-t. naj.
— pain about: naj.
— palpitation: apis. convol. lill-t. naj.
Heat, running into thigh: pod.
— flashes of: plat. sulph.
Hold to something, feels that she must: SEP.
Hunger, but little food distresses: lyc.
Hyperæsthesia of senses: therid.
Hypogastrium, soreness and heaviness in: helo.
Hysteria: absin. chenopod-v. cimi. ferr-iod. stram. therid. znc-val.
Iliac region, pain in: thuj.
Inguinal region, drawing in: *plat.*
Injuries from: ARN. con. *ham. hypericum.*
Irritability: cham. nux-v.
Labor, during: lill-t.
Leg, pain in: ars.
Leucorrhœa: iod. lill-t. thu. xanth.
— excoriating: arg-m. onos.
Leucorrhœa, offensive: arg-m.
— profuse: eupion.
— running down legs: alu. onos. SYPH.
— staining brown: lill-t. ustl.
— thick, yellow, burning: iod.
Liver, affections of: *pod.*
Lochia, suppressed: verat-v.
Loins, pain in: arg-m.
Lungs, sympathetic symptoms in: apis.
Lying down, necessity to be: thu.
— on back, pains as if broken when: *goss.*
— with knees to chin: lac-can.
Mammæ, atrophy of: IOD.
— heaviness, sense of in: IOD.
— pain in: lill-t.
— soreness in: lac-can.
Melancholy: cimi.
Menorrhagia: alet. crot-h. ham. helo. lach. lill-t. plat.
Menses, after: cup. graph. iod. znc.
— before: graph.
— beginning: bell. coccl.
— irregular: apis. guai.
— offensive: lach.
— retarded: xanth.
— scanty: lach. ovi-gal-p. ustl. xanth.
— suppressed: acon. cimi. graph. ustl.
— vicarious: *bry.* ham.
Metritis: alu. verat-v.
Metrorrhagia: apis. arg-n. ars. bell. lach. lill-t. ustl.
Mind, depression of: naja.
Navel to pelvis, pains extending from: *pallad.*
Neck, nape of, pains in: cimi. gels. onon.
Nervous symptoms: kali-br. syph. vibur. znc-val.
Numbness of extremities: aco. apis. ars. pod. sec-c.
Nymphomania: ant-c. coca. hedeoma. plat.
Obesity: graph.
Occiput, pains in: gels.
Pains wandering: goss. pul.
Palpitation: cac-gr. lill-t. naj.
Peritonitis: bell. bry. merc.
Ptosis: gels. sep.
Pregnancy: ham.
Pruritus vulvæ: kali-br. *tarent.*
Rectum, prolapsus of: pod.
Reflex symptoms numerous: plat.
Respiration, difficult: colo.
Restlessness: aco. *ars.* colo. lac-can.
Rheumatic affections: bry. cimi. guai. phyt. rhus-t.

CONCOMITANTS.

Sacrum, pains in: vespa.
Scapulæ, burning between: phos.
— weakness between: bursa-pas.
Screaming: atrop.
Scrofula: bar-m. cimi.
Sexual subjects, mind dwells on: staph.
Sexual instinct suppressed: coni. lach.
— — diminished: kali-br. onos.
— — excited: coca. plat.
Septicæmia: ars. CROT-H. *lach.* pyrogen. sulph.
Side, pain in: kali-ph.
Sighing: ign.
Skin dry: alu. artem-v.
— transparent: apis.
— waxy: ars.
Spleen, pain in: carb-v.
Sterility: bar-m. BELL. *brom.* canth. *coni.* eup-pur, helo. *iod.* plat. phos. phyt. plb. pul.
Strangury: canth.
Stretch limbs, desire to: cup. plb.
Suicide, contemplates: aur.
Sweat without relief: merc.
Sycosis: arg-n. thu.
Syphilis: ars. aur. kali-iod. *merc.* thu.
Tenesmus recti et vesica: merc.
Thirstlessness: apis. gels. puls.
Thighs, heat down: pod.
— drawing in anterior muscles of: vibur.
Tired, continually: thu.
Urinate, urging to: *sep.*
Urine frequent: apis. ferr-ph. vesp.
— profuse: apis.
— scanty: apis.
Uterus, bearing down in: ferr-ph.
— dropsy of: chin.
— hemorrhage from: amm-br. chin.
— pain in: pod.
— — — spasmodic: phos.
— polypi in: con.
— prolapsus of: arg-m. lill-t.
— weight in: carb-v.
Vagina dry: lyc.
— flatus from: brom. bursa-p.
— itching in: lill-t.
Vaginismus: aur.
Vertigo on rising: aco. bry. pho. ver-v. vibur.
Vomiting: colo.
Walk, is impelled to: fluor-ac. xanth.
Weakness: ars. dios.
Wet, getting feet: graph. pul.
Worm complaints: saba.

